# DXA, bioelectrical impedance, ultrasonography and biometry for the estimation of fat and lean mass in cats during weight loss

**DOI:** 10.1186/1746-6148-8-111

**Published:** 2012-07-10

**Authors:** Naida C Borges, Ricardo S Vasconcellos, Aulus C Carciofi, Karina N V Gonçalves, Francisco J A Paula, Daniel E Faria Filho, Júlio C Canola

**Affiliations:** 1 Veterinary Hospital, Veterinary and Zootechny School, Federal University of Goiás, Goias State, Brazil; 2 College of Agrarian and Veterinarian Sciences, University of Jaboticabal, São Paulo State, Brazil; 3 Faculty of Medicine of Ribeirão Preto, University of São Paulo, São Paulo State, Brazil; 4 Department of Zootechny, Federal University of Minas Gerais, Minas Gerais State, Brazil

## Abstract

**Background:**

Few equations have been developed in veterinary medicine compared to human medicine to predict body composition. The present study was done to evaluate the influence of weight loss on biometry (BIO), bioimpedance analysis (BIA) and ultrasonography (US) in cats, proposing equations to estimate fat (FM) and lean (LM) body mass, as compared to dual energy x-ray absorptiometry (DXA) as the referenced method. For this were used 16 gonadectomized obese cats (8 males and 8 females) in a weight loss program. DXA, BIO, BIA and US were performed in the obese state (T0; obese animals), after 10% of weight loss (T1) and after 20% of weight loss (T2). Stepwise regression was used to analyze the relationship between the dependent variables (FM, LM) determined by DXA and the independent variables obtained by BIO, BIA and US. The better models chosen were evaluated by a simple regression analysis and means predicted vs. determined by DXA were compared to verify the accuracy of the equations.

**Results:**

The independent variables determined by BIO, BIA and US that best correlated (p < 0.005) with the dependent variables (FM and LM) were BW (body weight), TC (thoracic circumference), PC (pelvic circumference), R (resistance) and SFLT (subcutaneous fat layer thickness). Using Mallows’Cp statistics, p value and *r*^*2*^, 19 equations were selected (12 for FM, 7 for LM); however, only 7 equations accurately predicted FM and one LM of cats.

**Conclusions:**

The equations with two variables are better to use because they are effective and will be an alternative method to estimate body composition in the clinical routine. For estimated lean mass the equations using body weight associated with biometrics measures can be proposed. For estimated fat mass the equations using body weight associated with bioimpedance analysis can be proposed.

## Background

The body composition is used to describe the percentages of fat, bone and muscle. Therefore, two people of equal height and body weight may look completely different from each other because they have a different body composition. Fat mass (FM) and lean mass (LM) can be estimated and determined by different techniques, varying in precision and accuracy. Methods described thus for dogs and cats include body mass index, body condition score (BCS), biometry (BIO), dilution methods, bioelectrical impedance analysis (BIA), ultrasonography (US) and dual energy x-ray absorptiometry (DXA) [1,2].

Many equations are available to estimate the body composition of humans. Due to the convenience of application, BIO is the non-invasive method most used to characterize groups and populations [3,4]. Regardless of the method used to predict the body composition, the validity of an equation depends on the degree of precision and accuracy with which the variables are estimated within or outside the population of origin [5]. Therefore, most of the equations are specific, and can achieve the highest predictive ability when applied to a population similar to that from which were derived [3,4].

Few equations have been developed in veterinary medicine compared to human medicine to predict body composition. Associating results of BIO and BIA, different predictive equations for total body water, body protein, LM and FM have been proposed [6]. For dogs and cats, equations for estimation of body composition using chemical analysis [7], biometry [8] and ultrasonography [2] have also been proposed. The BCS system, like the one proposed by Laflamme [9] still is the method most widely used by clinicians to monitor changes in body condition during obesity treatment in cats. However, practical equations, easy to use that allow a more accurate prediction of LM and FM than the BCS system could be of value during the evaluation and follow-up of nutritional interventions in cats.

Equations to predict body composition are very useful in terms of practical aspects, especially during nutritional interventions and evaluation of individual responses to nutritional therapy. Noninvasive reference methods, such as DXA or deuterium isotope dilution, although accurate and sensitive are not available in all research laboratories and are not feasible in most clinical practices. In these contexts US and BIA could represent less expensive and available equipment. The validity and utility of these instruments and methods, however, depend on finding equations that generate estimates of body composition from the variables generated by them. Such equations must be validated, and their accuracy and precision must be determined.

The objective of the present study was to evaluate the influence of weight loss on biometry, bioelectrical impedance and ultrasonography in cats, proposing equations to estimate fat and lean body mass, as compared to DXA as the referenced method.

## Results

### DXA, BCS, BIO, BIA and US

Body composition analyzed by DXA, and BCS, BIO, BIA and US measurement are described in Table 1. The independent variables determined by BIO, BIA and US that best correlated with the dependent variables (FM and LM) were BW, TC, PC, R and SFLT (p < 0.005).

**Table 1:** Means and standard deviation of body condition score (BCS), body composition analyzed by DXA and measurements by biometry (BIO), bioimpedance analysis (BIA) and ultrasonography (US) in males (M) and females (F) cats, before weight loss (T0), and after 10% (T1) and 20% (T2) of body weight loss

**Variables**	**Animals**	**T0**	**T1**	**T2**
BCS^1^	F*	8.7(0.5)	8.0 (0.8)	6.7 (1.1)
	M*	8.6 (0.5)	7.9 (0.7)	6.0 (0.8)
TBM (g)	F	4576.0 (914.7)	4005.0 (873.2)	3581.0 (770.0)
	M	5258.0 (1175.1)	4617.0 (1145.0)	4105.0 (1055.9)
FM(g)	F	1929.0 (540.3)	1430.0 (541.6)	1026.0 (371.5)
	M	1968.0 (675.0)	1426.0 (670.4)	1077.0 (534.3)
FM (%)	F	41.6 (4.0)	34.9 (7.4)	28.0 (4.9)
	M	36.7 (4.6)	29.7 (6.5)	25.3 (6.3)
LM (g)	F	2538.0 (383.1)	2474.0 (431.2)	2458.0 (425.0)
	M	3153.0 (512.9)	3063.0 (488.5)	2902.0 (586.7)
LM (%)	F	56.0 (4.0)	62.6 (7.3)	69.3 (4.8)
	M	60.7 (4.5)	67.5 (6.4)	71.6 (6.1)
BW (g)	F	4781.3 (839.3)	4228.8 (868.8)	3788.8 (769.8)
	M	5431.3 (1174.4)	4881.3 (1125.8)	4366.3 (1036.4)
BL (cm)	F	47.5 (4.0)	47.5 (4.0)	47.1 (3.6)
	M	50.5 (2.1)	50.8 (2.1)	49.9 (2.3)
TC (cm)	F	39.6 (2.4)	36.2 (4.4)	34.0 (3.2)
	M	41.3 (3.1)	38.0 (3.8)	35.6 (3.8)
PC (cm)	F	42.3 (3.3)	38.5 (4.9)	36.3 (4.1)
	M	46.5 (4.9)	39.8 (5.2)	36.9 (4.9)
RTL (cm)	F	16.9 (1.8)	16.9 (1.1)	16.7 (1.3)
	M	17.8 (1.7)	18.2 (1.6)	17.3 (1.8)
RPL (cm)	F	18.6 (1.8)	19.0 (1.6)	18.8 (1.6)
	M	19.3 (1.6)	19.7 (1.4)	19.3 (1.0)
SFLT (cm)	F	0.065 (0.02)	0.048 (0.01)	0.023 (0.01)
	M	0.071 (0.03)	0.048 (0.02)	0.020 (0.02)
R (Ω)	F	176.9 (14.3)	180.7 (13.2)	185.8 (21.5)
	M	167.3 (17.4)	162.9 (16.0)	182.1 (23.6)
Xc (Ω)	F	27.0 (3.5)	28.2 (3.1)	28.3 (4.0)
	M	28.5 (2.0)	23.3 (6.2)	24.9 (4.3)

*F (n = 8) and *M (n = 8); ^1^ according to Laflamme (1997).

BW, body weight; BL, body length; TC, thoracic circumference; PC, pelvic circumference; RTL, right thoracic limb length; RTL, right pelvic limb length; R, resistance; Xc, reactance; SFLT, subcutaneous fat layer thickness.

### Predictive equations for FM and LM

In order to correct factors that eventually might affect the precision and accuracy of the equations, all results of BIO, BIA and US were previously tested for homogeneity, linearity and multicolinearity [5,10]. The stepwise multiple regression analysis produced 32 equations, 24 for FM estimation and eight for LM estimation. The 19 better models (17 for FM and seven for LM) obtained are listed in Tables 2 and 3.

**Table 2:** **Equations to predict cat fat mass and stepwise multiple regression (adjusted coefficient of determination (*r***^***2***^**), Mallows’Cp statistic (Cp), statistical significance (p) and root mean square error (RMSE))**

	**Equations for fat mass estimation (kg)**	**Equation number**	***r***^***2***^	**Cp**	**p**	**RMSE (kg)**
M (n = 24)	0.7BW + 3.22PC/TC–0.005BW/RTL-4	1	0.94	1.23	=0.04	0.19
F (n = 24)	−0.07BL + 0.9BW + 0.008R - 0.60	2	0.93	1.97	=0.01	0.17
	0.4BW + 11.50 SFLT-0.69	3	0.94	3.46	=0.01	0.16
M and F (n = 48)	0.4BW + 0.006R + 9.67 SFLT-1.84	4	0.90	1.25	=0.009	0.21
	−0.05BL + 0.7BW + 0.007R - 0.60	5	0.88	1.95	=0.009	0.24
	0.3BW + 9.97 SFLT-0.57	6	0.88	2.90	=0.009	0.23
T0 (n = 16)	0.5BW + 0.007R - 1.88	7	0.86	6.50	=0.05	0.25
T1 (n = 16)	0.4BW + 0.01R + 16.13 SFLT-2.71	8	0.93	1.20	=0.009	0.17
	0.6BW + 0.01R-2.84	9	0.86	1.01	=0.04	0.24
	0.3BW + 15.49 SFLT-0.69	10	0.86	0.75	=0.04	0.24
T2 (n = 16)	0.5BW + 3.55PC/TC–0.005BW/RTL + 8.48 SFLT-3.74	11	0.94	5.00	=0.02	0.12
	−0.022BL + 0.7BW + 4.24PC/TC–0.006BW/RTL-3.65	12	0.94	4.50	=0.02	0.13

M, males; F, females; BW, body weight; T0, (time zero – obese animals); T1, (time one −10% reduction in BW); T2 (time two – 20% reduction in BW); BL, body length; PC, pelvic circumference; TC, thoracic circumference; RTL, right thoracic limb length; R, resistance; SFLT, subcutaneous fat layer thickness.

**Table 3:** **Equations to predict cat lean mass and stepwise multiple regression (adjusted coefficient of determination (*r***^***2***^**), Mallows’Cp statistic (Cp), statistical significance (p) and root mean square error (RMSE))**

	**Equations for lean mass estimation (kg)**	**Equation number**	***r***^***2***^	**Cp**	**p**	**RMSE (kg)**
M (n = 24)	0.3BW + 3.0PC/TC – 0.003BW/RTL + 3.51	13	0.92	2.31	=0.04	0.16
F (n = 24)	−0.003BW/RTL + 0.11BL^2^/R + 0.40	14	0.83	0.45	=0.04	0.17
M and F (n = 48)	0.2BW + 0.09BL^2^/R + 0.25	15	0.85	4.72	=0.0001	0.21
	0.04 BW + 0.07 BL^2^/R – 3.7SFLT + 0.17	16	0.87	2.65	=0.05	0.20
T0 (n = 16)	0.4BW + 0.08BL^2^/R - 0.05	17	0.86	4.23	=0.05	0.22
T1 (n = 16)	0.3BW + 0.08BL^2^/R + 0.20	18	0.87	6.40	=0.02	0.17
T2 (n = 16)	0.3BW – 2.71PC/TC – 0.004BW/RTL + 0.05BL^2^/R + 2.73	19	0.97	3.09	=0.02	0.10

M, males; F, females; BW, body weight;T0, (time zero – obese animals); T1, (time one – 10% reduction in BW); T2 (time two – 20% reduction in BW); BL, body length; PC, pelvic circumference; TC, thoracic circumference; RTL, right thoracic limb length; BL^2^/R, impedance index; R, resistance; SFLT, subcutaneous fat layer thickness.

Separating data according to gender, for FM (Table 2) the association of biometric measurements accounted for 94% of the variation in FM content of males (equation 1; Cp = 1.23; p < 0.05). For females two equations for FM estimation were chosen, number 2 (*r*^*2*^ = 0.93; Cp = 1.97; p < 0.01) and number 3 (*r*^*2*^ = 0.94; Cp = 3.46; p < 0.01).

When data were separated by time of evaluation six equations were proposed (Table 2), one using BW and R (*r*^*2*^ = 0.86, equation 7), one using BIO, US and BIA all together (equation 8), one using the combination of BIO and US (*r*^*2*^ = 0.94, equation 11), and three using only one measure, which are BIA (*r*^*2*^ = 0.9486, equation 9), US (*r*^*2*^ = 0.86, equation 10) and BIO (*r*^*2*^ = 0.94, equation 12). Finally, analysis independent of gender and obese state (*n* = 48) resulted in three equations to estimate FM, one using BIO, US and BIA (*r*^*2*^ =0.90, equation 4), one using BIO and BIA (*r*^*2*^ = 0.87, equation 5), and one using BIO and US (*r*^*2*^ = 0.88, equation 6).

For LM prediction (Table 3), the association of biometric measurements accounted for 92% of the variation for males (equation 13; Cp = 2.31; p = 0.04). Regarding females the better model obtained was described by equation 14 (*r*^*2*^ = 0.83; Cp = 0.45; p = 0.04). The analysis independent of gender and obese state (*n* = 48) resulted in two equations to predict LM, one with a combination of BW and the impedance index, in other words, BL^2^/R (*r*^*2*^ = 0.85, equation 15), and the other with the BW, impedance index and SFLT (*r*^*2*^ = 0.87, equation 16).

**Figure 1:**
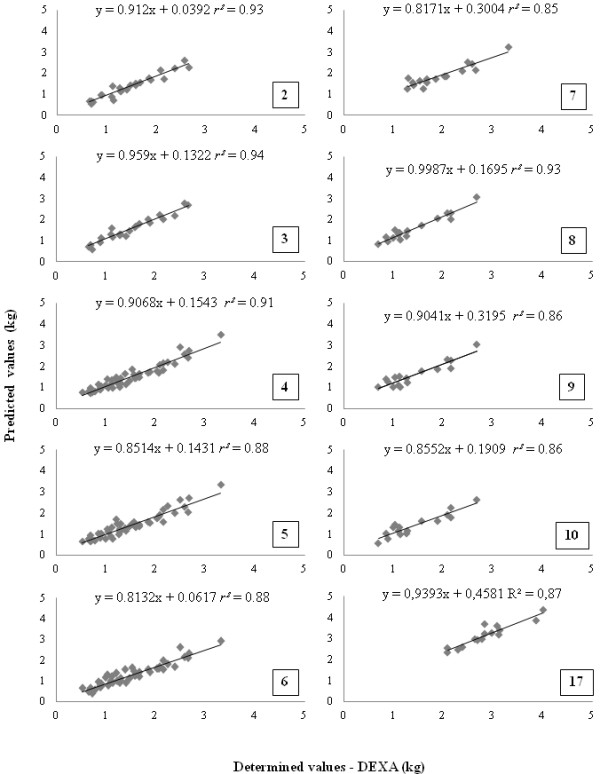
Regression analysis of determinate (by DXA) vs. predicted values by the equations 2, 3, 4, 5, 6, 7, 8, 9 and 10 for fat mass (FM) and equation 17 for lean mass (LM) in cats.

The comparisons between DXA results and values predicted by the equations are listed in Table 4. For FM, similar results were obtained between DXA and estimations generated by equations 2 to 10 (p > 0.05). For LM estimation, only the result of equation 17 was similar to DXA (p > 0.05). The results of these equations are illustrated in Figure 1.

**Table 4:** Comparison of fat mass (FM) and lean mass (LM) determined by DXA and estimated by the equations 1 to 19 (means and SD) in cats

	**DXA (kg)**	**Estimated values (kg)**	**Equation number**	**p value (*t*-Test)**	**Linear regression**	***r***^***2***^
**FM**						
M (*n* = 24)	1.49 (0.71)	2.88 (0.94)	1	<0.0001	y = 0.728x - 0.605	0.93
F (*n* = 24)	1.46 (0.60)	1.37 (0.57)	2	= 0.60	y = 1.014x + 0.069	0.92
		1.53 (0.59)	3	= 0.67	y = 0.976x - 0.036	0.94
M and F (*n* = 48)	1.48 (0.65)	1.49 (0.62)	4	= 0.90	y = 1.002x - 0.019	0.91
		1.40 (0.59)	5	= 0.55	y = 1.034x + 0.028	0.88
		1.26 (0.56)	6	= 0.09	y = 1.086x + 0.105	0.88
T0 (*n* = 16)	1.95 (0.59)	1.89 (0.52)	7	= 0.78	y = 1.042x - 0.023	0.85
T1 (*n* = 16)	1.43 (0.45)	1.60 (0.61)	8	= 0.43	y = 0.932x - 0.058	0.93
		1.61 (0.58)	9	= 0.38	y = 0.945x - 0.094	0.85
		1.41 (0.54)	10	= 0.94	y = 1.004x + 0.010	0.86
T2 (*n* = 16)	1.05 (0.45)	2.21 (0.61)	11	<0.0001	y = 0.691x - 0.473	0.89
		2.59 (0.68)	12	<0.0001	y = 0.612x - 0.535	0.87
**LM**						
M (*n* = 24)	3.04 (0.52)	8.19 (0.48)	13	<0.0001	y = 0.882x - 4.179	0.67
F (*n* = 24)	2.49 (0.40)	1.79 (0.27)	14	<0.0001	y = 1.299x + 0.168	0.79
M and F (*n* = 48)	2.76 (0.53)	1.59 (0.25)	15	<0.0001	y = 1.868x - 0.202	0.75
		1.15 (0.19	16	<0.0001	y = 2.047x + 0.402	0.52
T0 (*n* = 16)	2.85 (0.54)	3.13 (0.55)	17	= 0.15	y = 0.925x - 0.051	0.87
T1 (*n* = 16)	2.77 (0.54)	1.68 (0.36)	18	<0.0001	y = 2.008x - 0.210	0.78
T2 (*n* = 16)	2.68 (0.54)	0.44 (0.15)	19	<0.0001	y = 2.651x + 1.509	0.56

M, males; F, females; T0 (before weight loss [WL]); T1 (after 10% of WL); T2 (after 20% of WL); y = estimated value; x = determined value.

## Discussion

### DXA, BCS, BIO, BIA and US

The differences in body composition observed between genders and after weight loss confirm data reported by several authors in studies on humans [11,12] and animals [6,13-15]. These data support the need for studies of body composition considering the influence and importance of breed, sex, and sexual condition, allowing a better understanding the influence of these factors on cat body composition. Considering that males had lower FM (%) them females, we can conclude that TC and PC values do not necessarily reflect the FM (%) of a particular cat. These results must consider the gender.

The current study confirmed that the 9-point BCS [9] correlated well with body fat mass estimated by DXA. The main value of this score system is that it helps clinicians and owners to estimate the ideal body composition for their pets. However, a high coefficient of variation (from 13.9% for BCS 9 to 25.8% for BCS 5) between BCS and FM (%) was verified, demonstration the need for more precise procedures for nutritional studies.

The US measurement of the SFLT proved to be a sensitive method since an important reduction of subcutaneous fat was verified during weight loss. SFLT results alone also presented a good correlation with DXA results of FM. However, the results obtained here could not be compared because this method was not utilized in the consulted bibliography. Ultrasonography is of simple application, is available in clinical practice, and can be used to monitor changes in body composition during nutritional intervention. However, future studies are necessary to validate it.

### Predictive equations for FM and LM

Adopting the criteria used by MacNeil [16], Guo et al. [5,11] and Freund and Littell [10] for the selection of predictive models, i.e., statistical significance of the independent variables (p < 0.05), high coefficient of determination (*r*^*2*^), and lower Mallows’Cp statistics and RMSE values, 19 equation for FM and LM prediction were selected.

Different equations were generated for males and females. The suggested equation for males used only biometric measurements, while BIA and US proved to be valuable for females. The amount of body fat mass also influenced the equations suggested for FM and LM estimations. BIA was valuable for more obese animals, while biometry was important for less fat animals. Some of the equations generated in the present study, however, are too complex for practical use. Equations 4, 8 and 16, for example, were selected in the mathematical process but use three types of animal evaluation (biometry, BIC and US).

Although several equations resulted in FM and LM values statistically similar to that determined by DXA, only the equations 4, 5, 7, and 10 resulted in means that differed less than 5% from the DXA values. Equation 4 is very complicated to be used in practice; however, for research purposes it could be sensitive enough to understand changes in body composition during diet or protocols for weight loss evaluations in cats. Equation 5 appears to be the most interesting for FM estimations in practice, using simple biometry and BIA. For obese cats, FM could also be estimated with equation 7, again using body weight and BIA.

Several studies on human beings also reported BIA as a good technique for the prediction of FM [17,18]. For humans, BIA is also suggested as a good method for LM [3] estimations, which were not verified for cats in the present study. For LM estimations, no equation could be found in the present assay (considering that equation 17 resulted in a mean LM value approximately 10% greater than the DXA results). Controversies about the use of BIA for LM estimation for humans, however, still exist [4,19].

The independent variable SFLT was used in only one equation for LM estimation, i.e. equation 16. However, the result obtained was 17% percent lower than the DXA result. This was surprising because BIA, whose results did not change with weight loss, was more correlated with body FM estimations than SFLT, which presented a significant reduction during the weight loss. Anyway, the measurement of SFLT appears to be an interesting variable to study in other experiments about the prediction of cat body composition.

In the present study the equations were developed and further tested in the same animals to assess their accuracy. Several measurements were made on the same animals along the process of weight loss. If, on the one hand, this allowed an understanding of the changes in the variables studied regarding the weight loss of cats, we must consider that the use of the same animals may have influenced the results, a fact that should be considered with caution. Another important aspect to consider during the process of development and validation of predictive equations is the introduction of age ranges, weight ranges, height ranges, sexual status, and breeds. Compared with human beings, few studies have been conducted with this purpose in companion animals.

## Conclusions

The equations with two variables are better to use because they are effective and will be an alternative method to estimate body composition in the clinical routine. For estimated lean mass the equations using body weight associated with biometrics measures can be proposed. For estimated fat mass the equations using body weight associated with bioimpedance analysis can be proposed.

## Methods

### Animals and experimental design

All experimental protocols were approved by the animal welfare and use committee of the College of Agrarian and Veterinarian Sciences, São Paulo State University, Brazil.

Sixteen gonadectomized mongrel adult cats, eight males and eight females, were selected. The average of body weight (BW) was 4.8 ± 0.8 kg and 5.5 ± 1.1 kg, respectively, for females and males. Obesity was considered to be present when cats had a BCS of at least 8 on a 9 points scale [9]. The cats were kept in individual cages (0.8 x 0.8 x 0.8 m) for 14 hours (from 18:00 h to 08:00 h) and then released during the rest of the day into a collective area (36 m^2^) for exercise and socialization. Food was offered at 18:00 h and any remaining food was removed at 08:00 h. Throughout the study, mean ambient temperature was 21.75 ± 0.8 °C and a 12 h dark:12 h light cycle was provided.

The diet fed for weight loss presented, per M Joule of metabolizable energy: 28.4 g crude protein, 6.4 g of ether extract, 9.7 g of total dietary fiber, 16.1 g of starch, 4.6 g of ash, 0.9 g of calcium, and 0.7 g of phosphorus. To achieve weight loss, cats were fed 60% of their estimated maintenance energy requirements, calculated according to the NRC equation for obese cats [20], which equaled 326 MJ (body weight, in kg)^0.4^. Cats were weighed weekly, at the same time of day using a digital scale (Digital Weight Scales, model LC 50, Marte, São Paulo, SP). Under this management, all cats lost approximately 20% of their initial body weight in 24.6 ± 0.25 weeks.

During the study cats were evaluated at three times: obese state (T0; obese animals), middle of weight loss (T1; body weight [BW] 10% lower than T0), and end of weight loss (T2; BW 20% lower than T0). The results obtained at the three times were analyzed statistically considering three possibilities: cats independently of gender and obese state (*n* = 48), males or females (*n* = 24) independently of time and considering the time of evaluation (T0, T1 or T2), independently of gender (*n* = 16). These data were used to produce specific predictive equations for body composition.

### Body measures

Biometry (BIO), BCS, BIA, US and DXA exams were performed in triplicate at T0, T1 and T2. Before each exam, cats were fasted for 12 hours and then anesthetized with a combination of levomepromazine (Neozine 5 mg/ml, Aventis Pharma LTDA, São Paulo, SP), tiletamine and zolazepam hydrochloride (Zoletil 50 mg/ml, Virbac do Brasil Indústria e Comércio LTDA, São Paulo, SP) administered intramuscularly at respective doses of 0.5, 2.5 and 2.5 mg/kg. After loss of the postural reflex, cats were positioned according to the requirements of each technique.

Body composition was determined by DXA (QDR 4500 Elite Windows®, Guide Hologic Inc. 35, Bedford, MA, USA) with three consecutive scans without repositioning the animals between scans, as described by Lauten et al. [21,22]. The whole body images were analyzed using pediatric software (Pediatric whole body uses QDR for Windows® 98 System, Guide Hologic Inc. 35. Bedford, MA, USA). The precision of this method was determined [23] before this study and the coefficient of variation ranged from 3.2% to 4.3% for LM and from 7.7% to 10.9% for FM.

The cat’s BCS and BIO classification during the study was done by the same trained veterinarian (VASCONCELLOS, R.S) [24]. BIO measurements were made using a metric tape with centimeter divisions, with the animal positioned in left lateral decubitus as described by Stanton et al. [6]. Body length was measured from the nostrils to the sacrococcygeal joint, accompanying the dorsal line of the animal. The pelvic circumference was measured over the last lumbar vertebra [6] and the thoracic circumference was measured over the 7^th^ intercostals space [8]. The right pelvic limb length was measured from the right coxofemoral joint to the lateral malleolus of fibula and the right thoracic limb length was measured from the scapulohumeral joint to the ulnar tubercle [6].

The BIA exam was performed using a monofrequential Bioimpedance Analyzer (Quantum II, model RJL, RJL Systems Inc., Clinton, MI, USA). Four acupuncture needles (0.40 x 15 mm) with a spiral head covered with copper coupled to the connection clamp were used as electrodes. The positioning of the animals and the points of needle insertion were based on the methodology described by Stanton et al. [6]. Resistance (R) and reactance (Xc) were measured in three consecutive readings, with the apparatus being disconnected between measurements. The phase angle (PA) was calculated by the arc-tangent relation of R and Xc (PA = arctan Xc/R). The result of PA, expressed as radians, was converted to degrees by multiplying the value by 1800/π or 57.296.

Ultrasonography was performed with ultrasound equipment (Pie Medical Scanner 200 C, model 41480, Can Medical, Kingston, ON, Canada) using a multifrequential linear arrangement transducer of 6 to 8 MHz, with electronic scanning being carried out at the highest frequency. The transducer was covered with gel on its transmitting and receiving surfaces, inserted into a specifically made silicone cushion and positioned transversely on the region corresponding to the seventh lumbar vertebra, as described by Morooka et al. [25].

**Figure 2:**
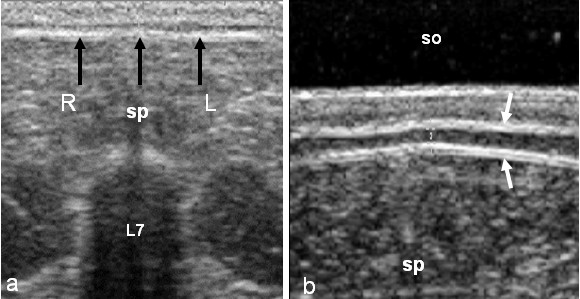
**Ultrasonogram of the measurement of fat layer.** (a) Transversal sonogram demonstrates the places (arrows) to measurements of fat layer. (sp) spinous process of 7th lumbar vertebrae (L7), (R) right side, (L) left side. (b) Image in detail with the use of standoff (so). The fat layer is a hypoechoic line between two hyperechoic lines (arrows). (sp) spinous process

The fat layer, a hypoechoic line between two hyperechoic lines (skin and subcutaneous connective tissue) was measured in centimeters. The probe was first placed on the top of the spinous process of L7 and then was moved horizontally to the right and left (Figure 2).

### Statistical analysis

DXA, BCS, BIO, BIC, and US results are reported as means plus standard deviation (*s*). Pearson’s correlations were used to determine the significance of associations among the body measure variables. All statistical analyses were performed using the SAS® program (PROC GLM, SAS Institute Inc., Cary, NC) with the level of significance set at 5%.

Stepwise multiple regressions were used to elaborate equations to estimate the dependent variables FM and LM, according to method proposed by Freund and Littell [10]. The considered independent variables were body weight, body length, right pelvic and thoracic limbs, pelvic and thoracic circumference, resistance, reactance, and subcutaneous fat layer thickness. The impedance index BL^2^/R was included as an independent variable as recommended by Kushner et al. [19] to estimate LM. This methodology was used to propose models for male cats (*n* = 24), female cats (*n* = 24), for the two gender together (*n* = 48), and for cats before and after weight loss (*n* = 16).

The general linear regression model used was y = β _0_ + β _1*_ x_1_ + … + β _m*_x_m_ + ϵ, where y represents the dependent variable; β _0,_ β _1_,… β _m_ represent the unknown parameters; x_1_*x*_2_ + … + x_m_ the prediction variables; and ϵ the random error [10]. For the execution of regression analysis, the factors that interfere with the precision and accuracy of the predictive equations were tested and controlled as cited by Guo et al. [5] and Freund and Littell [10]. Multicolinearity was detected and the parameters involved were PC and TC. To correct the problem two indexes were generated (PC/TC and BW/RTL) and replaced these variables in the regression analysis.

The better models obtained were selected according to the criteria cited by MacNeil [16], Guo et al. [5,11], and Freund and Littell [10], i.e., significance of the independent variables (p ≤ 0.05), highest coefficient of determination (*r*^*2*^), lowest Mallows’Cp statistic and the association of these parameters with the practical quality of the model. The Mallows’Cp statistic was calculated according to the following mathematical model: Cp = *SS*_*e*_*/RMS – (n - 2p)*, where *SS*_*e*_ is the residual sum of squares, *RMS* is the residual mean square of the model, *n* is the number of observations, and *p* is the number of independent variables [16].

After choosing the better models for FM and LM predictions, the average results of DXA were compared with the average predicted results obtained by the equations generated by the model. These comparisons was made using the *t*-Test (p < 0.05) and simple regression analysis (PROC REG, SAS Institute Inc., Cary, NC) between the values estimated by the equations and determined by DXA.

## Abbreviations

BCS, Body condition score; BIA, Bioimpedance analysis; BIO, Biometry; BL, Body length; BL2/R, Impedance index; BW, Body weight; Cp, Mallows’Cp statistic; CV, Coefficient of variation; DXA, Dual energy x-ray absorptiometry; F, Females; FM, Fat mass; LM, Lean mass; M, Males; p, Statistical significance; PA, Phase angle; PC, Pelvic circumference; R, Resistance; r2, Coefficient of determination; RMSE, Root mean square error; RTL, Right thoracic limb length; SFLT, Subcutaneous fat layer thickness; s, Standard deviation; T0, Time zero – obese animals; T1, Time one – animals after 10% reduction in BW; T2, Time two – animals after 20% reduction in BW; TC, Thoracic circumference; US, Ultrasonography; Xc, Reactance.

## Competing interests

The author(s) declare that they have no competing interests.

## Authors’ contributions

NCB and RSV planned and designed the study, performed the experiments and drafted the manuscript; the nutrition part of the experiment was designed by ACC, who have also, performed the experiments, drafted the manuscript and coordinated the study; KNVG helped performing the experiments; FJAP read, analyzed and performed the DXA; DEFF performed the procedures and the statistical analysis; JCC coordinated the study and helped to draft the manuscript. All the authors read and approved the final manuscript.
